# Raw Data to Results: A Hands-On Introduction and Overview of Computational Analysis for Single-Molecule Localization Microscopy

**DOI:** 10.3389/fbinf.2021.817254

**Published:** 2022-02-01

**Authors:** Koen J. A. Martens, Bartosz Turkowyd, Ulrike Endesfelder

**Affiliations:** ^1^ Department of Physics, Carnegie Mellon University, Pittsburgh, PA, United States; ^2^ Institute for Microbiology and Biotechnology, Rheinische-Friedrich-Wilhelms-Universität Bonn, Bonn, Germany; ^3^ Department of Systems and Synthetic Microbiology, Max Planck Institute for Terrestrial Microbiology, LOEWE Center for Synthetic Microbiology (SYNMIKRO), Marburg, Germany

**Keywords:** SMLM Python and MATLAB code, temporal median filtering, SMLM localization and localization merging, drift and chromatic aberration correction, SMLM image formation, single-particle tracking, SMLM clustering, SMLM localization precision and structural image resolution

## Abstract

Single-molecule localization microscopy (SMLM) is an advanced microscopy method that uses the blinking of fluorescent molecules to determine the position of these molecules with a resolution below the diffraction limit (∼5–40 nm). While SMLM imaging itself is becoming more popular, the computational analysis surrounding the technique is still a specialized area and often remains a “black box” for experimental researchers. Here, we provide an introduction to the required computational analysis of SMLM imaging, post-processing and typical data analysis. Importantly, user-friendly, ready-to-use and well-documented code in Python and MATLAB with exemplary data is provided as an interactive experience for the reader, as well as a starting point for further analysis. Our code is supplemented by descriptions of the computational problems and their implementation. We discuss the state of the art in computational methods and software suites used in SMLM imaging and data analysis. Finally, we give an outlook into further computational challenges in the field.

## Introduction

Single-molecule localization microscopy (SMLM) is a collective term for microscopy techniques that generate localization data of individual fluorescent molecule emission events, and can achieve ∼5–40 nm resolution at ∼10–100 Hz (Betzig et al., 2006; Rust et al., 2006; Sage et al., 2019). Localization-based microscopy can be performed with relatively standard, albeit sensitive, wide-field fluorescence microscopes. The key requirement is that the fluorescent molecules used are able to switch between *on* and *off* states, ensuring that all molecules are read out individually (Endesfelder et al., 2011). dSTORM (direct stochastic optical reconstruction microscopy) achieves this *on/off*-switching *via* chemical equilibria of organic fluorophores, often assisted *via* (near-)UV light and/or reactive chemicals (Rust et al., 2006; Heilemann et al., 2008). For *in vivo* SMLM imaging, PALM (photo-activated localization microscopy) is a conceptually similar technique as dSTORM, but relies on photo-induced chemical transitions of fluorescent proteins (Betzig et al., 2006; Manley et al., 2008). Alternatively, the *on/off*-switching can be accomplished by repetitive binding/unbinding of the fluorophore as done by PAINT microscopy (points accumulation for imaging in nanoscale topography) (Sharonov and Hochstrasser, 2006). As long as the fluorophore is unbound, it diffuses too rapidly to produce a well-formed point-spread function (PSF). This binding/unbinding is often, but not exclusively, induced via DNA complementarity, i.e. DNA-PAINT (Schnitzbauer et al., 2017).

A further increase in spatiotemporal resolution can be achieved by various improvements in sample, fluorophores, instrument, or computational design. For instance, increasing labeling density and specificity, increasing emitter fluorescence, or decreasing the distance between fluorophore to structure of interest will result in a better observed resolution (Grimm et al., 2016; Virant et al., 2018; Vojnovic and Endesfelder, 2020; Geertsema et al., 2021). Accurate axial drift correction and experimental PSF descriptions also have an influence (Li et al., 2018; Vojnovic and Endesfelder, 2020). Instrumentally, the *on/off*-switching of organic fluorophores or photo-activatable fluorescent proteins can be combined with structured illumination profiles, reaching up to 2–3 nm spatial resolution (Balzarotti et al., 2017; Gu et al., 2019; Cnossen et al., 2020; Jouchet et al., 2021).

All SMLM methods fundamentally result in an identical output: a movie of individual fluorophore emissions from which a coordinate list, containing at least time, *x*, and *y* positions of individual emitters, often complemented by information on localization uncertainty, emitter intensity, and axial (*z*) position, can be extracted. This output can principally be used to explore two main avenues: super-resolution imaging or single-particle tracking (spt).

In super-resolution imaging, the sample of interest is usually chemically fixed. Resolving all fluorophores’ positions, the fluorescently-tagged structure of interest can be visualized with a resolution about 10–20-fold lower than the classical diffraction limit [∼250 nm (Abbe, 1873)]. With the help of super-resolution imaging several unknown molecular arrangements in structural biology could be revealed and quantified and many review articles summarize these findings and achievements in detail (Huang et al., 2009; Patterson et al., 2010; Turkowyd et al., 2016; Baddeley and Bewersdorf, 2018; Sigal et al., 2018).

Alternatively, in spt, a natural biological sample (i.e. single living cells) with fluorophore-tagged proteins of interest are imaged (Manley et al., 2008). The behaviour of the individual intracellular biomolecules can be quantified, providing detailed information on molecular dynamics and interactions (Shen et al., 2017; Kapanidis et al., 2018; Elf and Barkefors, 2019). Spt can also be applied in *ex vivo* settings, such as membrane proteins in synthetic membranes or material science (Schütz et al., 1997; Martens et al., 2020).

Clearly, applications of SMLM imaging are highly diverse. Nevertheless, all of them inherently make use of similar computational analysis tools - from localization software, drift correction or color channel overlays to clustering or tracking routines. Over the past decades, a multitude of analysis methods and tools for localization data have evolved. Understanding the obligate computational details of SMLM imaging and knowing which tools to apply (when), and how to expand or modify them for a specific use case can be overwhelming, especially for researchers without a background in computer science. In this manuscript, we provide an overview of the most common computational analysis procedures in single-molecule localization microscopy and supply code written in Python and MATLAB. The structure of this work focuses on understanding of the problems and their solutions, rather than providing the most efficient or theoretically best solution. Wherever possible, information about less intuitive, but state-of-the-art alternatives is provided, as well as references to relevant software suites.

## Materials and Methods

### Samples

The *E. coli* RNA polymerase (RNAP) data for fiducial drift correction, image generation, clustering and Nearest Neighbor based Analysis (NeNA, (Endesfelder et al., 2014)) were taken from our previous work (Virant et al., 2017). Briefly, RNAPs were tagged with mEos3.2-A69T at their β′-subunit. Red, photoconverted mEos3.2-A69T fluorescence was read out using primed photoconversion. Movies were recorded with 16.67 Hz image acquisition until no new spots appeared. Localizations were obtained using RapidSTORM (Wolter et al., 2012).

The vimentin-BC2-tag data for the Fourier Ring Correlation (FRC) analysis were taken from our previous work (Virant et al., 2018). Briefly, vimentin, transiently expressed from a plasmid in HeLa cells, was tagged by the BC2 peptide tag sequence. After chemical fixation, cells were stained with the bivalent anti-BC2 nanobody labeled by AlexaFluor 647. The region of interest was imaged for 20,000 frames using the dSTORM imaging buffer (van de Linde et al., 2011) and localizations were obtained using RapidSTORM.

DNA-PAINT nanoruler SMLM data was recorded for the temporal median filter, localization, chromatic aberration and cross correlation drift correction modules. The GATTA-PAINT 80RG nanoruler was obtained from Gattaquant, Germany. 10.000 frames were recorded with 100 ms interval under 561 nm (1.5 kW/cm^2^) and 640 nm (1 kW/cm^2^) excitation, using a ZET405/488/561/640m dichroic, ZT405/488/561/640rpc rejection filter, and respectively ET610/75 or ET655LP bandpass filter.

For the single-particle tracking analysis, we prepared 20 nm diameter red and 200 nm diameter dark-red fluorescent beads (FluoSphere Thermo Fisher; 580 nm excitation/605 nm emission and 660 nm excitation/680 nm emission, respectively) in respectively a 1:1,000 and 1:10,000 dilution from the original stock in milli-Q water. ∼10 µL solution was placed on a coverslip and covered with another coverslip. Coverslips were gently pressed together to remove excess liquid and air bubbles and placed on the microscope. 10,000 frames were recorded with 15 ms interval, and 1 ms stroboscopic 488 and 561 nm laser illumination set at 3 and 0.3 kW/cm^2^, respectively. No bandpass filter was used. Localizations were obtained using ThunderStorm (Ovesny et al., 2014).

All movie and localization datasets used in the computational modules can be found on https://github.com/Endesfelder-Lab/SMLMComputational.

### SMLM Imaging

Imaging was performed on a custom build setup based on an automated Nikon Ti Eclipse microscope equipped with appropriate dichroic and filters (ET dapi/Fitc/cy3 or ZET405/488/561/640m dichroic, ZT405/488/561rpc or ZT405/488/561/640rpc rejection filter, ET610/75 or ET655LP bandpass, all AHF Analysentechnik, Germany), and a CFI Apo TIRF ×100 oil objective (NA 1.49, Nikon). The 488 nm, 561 nm, and 637 nm lasers (Coherent) was modulated via an acousto-optical tunable filter (AOTF) (Gooch and Housego, United States). Fluorescence was detected by an emCCD (iXON Ultra 888; Andor, United Kingdom). The *z*-focus was controlled by a commercial perfect focus system (Nikon, Germany). The sample was placed on a heating stage and kept at the constant temperature 25°C. Acquisitions were controlled by μManager (Edelstein et al., 2010).

### Code

**SCHEME 1:**
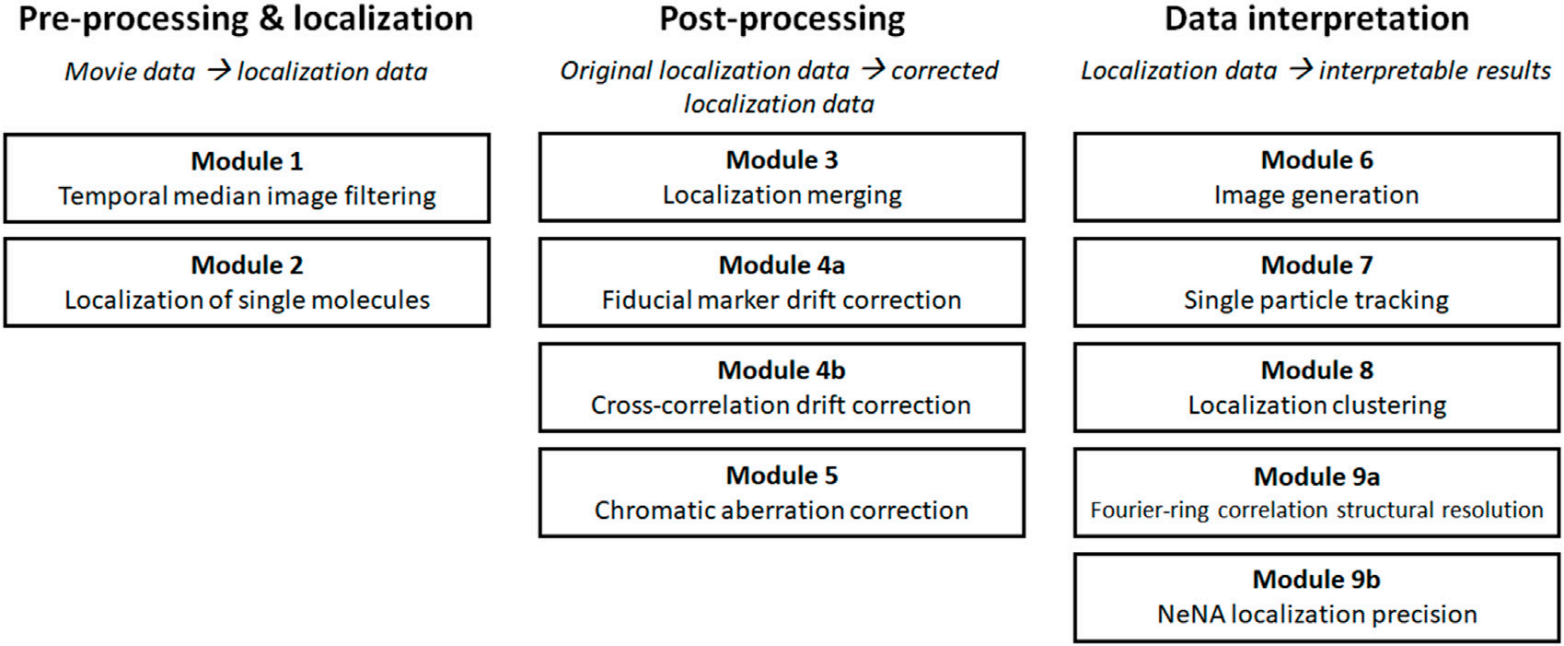
Overview of the modules.

All code, sub-divided into modules (Scheme 1) is provided both as Python code and as MATLAB code (https://drive.google.com/drive/u/0/folders/1lOKvC_L2fb78--uwz3on4lBzDGVum8Mc and https://github.com/Endesfelder-Lab/SMLMComputational) and is further documented by Pseudo-code (Supplementary information) and descriptions in the main text. The interactive environment of the google colab implementation (https://colab.research.google.com/) allows for direct, user-based testing and adaptation on our example data.

## Results

SMLM data is typically analyzed in several, mostly consecutive steps. The different analysis procedures in this manuscript follow this workflow are thus subdivided in three major groups: “pre-processing and localization”, “post-processing”, and “data interpretation” modules (Scheme 1). The modules of the first group “pre-processing and localization” all work on SMLM movie data and concern analysis steps which are used to properly translate the recorded movie material into localization data. In the second group, called “post-processing”, those raw localization lists are typically further refined in several routines that raise the quality of the data or combine different parts of data into final SMLM localization lists. These data sets then are visualized, characterized and interpreted by analysis routines which are grouped in “data interpretation”, and provide additional data (images, parameters, bionumbers and measurements etc.,) as output.

The order of our modules follows standard analysis practices, but some modules can be skipped or performed in a different order, and two modules (4 and 9) are subdivided into variant a and b as they present alternatives for similar tasks (i.e. drift correction and determination of structural resolution or localization precision). For every module, well-documented Pseudo-code, Python code, and MATLAB code is provided (https://drive.google.com/drive/u/0/folders/1lOKvC_L2fb78--uwz3on4lBzDGVum8Mc and https://github.com/Endesfelder-Lab/SMLMComputational, Supplementary Pseudocode S1–S9), which is accompanied by explanatory text and illustrations, as well as software alternatives, in the following text. We stress that this code is designed as “teaching material” rather than best-practice software, especially relating to speed optimization. An overview of existing SMLM analysis software that implement at least one of this manuscript’s modules is presented in Supplementary Table S1.

We encourage users of the codebase to not only run the analysis with the provided raw data, but also to apply it on their own data, and change the code accordingly and appropriately. To further assist users new to programming language, we have included a Supplemental Code environment (https://colab.research.google.com/drive/1Ht-WL-W3tpFfavDMZjDofLOR9HKP-nVV), where we show how to perform basic data handling, and include region-of-interest selection, pixel size conversion and intensity level correction. These little helper code snippets can be easily combined with the modules to filter, select and modify raw data as input for the chosen module.

Within our modules we focus on intuitive solutions to common SMLM analysis routines, and references to e.g. more complex or less intuitive state-of-the-art alternatives are provided. Specific analysis routines for highly specialized tasks - mostly for the third module group “data interpretation”- are out of scope of this work. These analysis procedures are not covered within our modules but the interested reader is pointed towards them in the discussion.

### Module 1: Temporal Median Image Filtering

**FIGURE 1:**
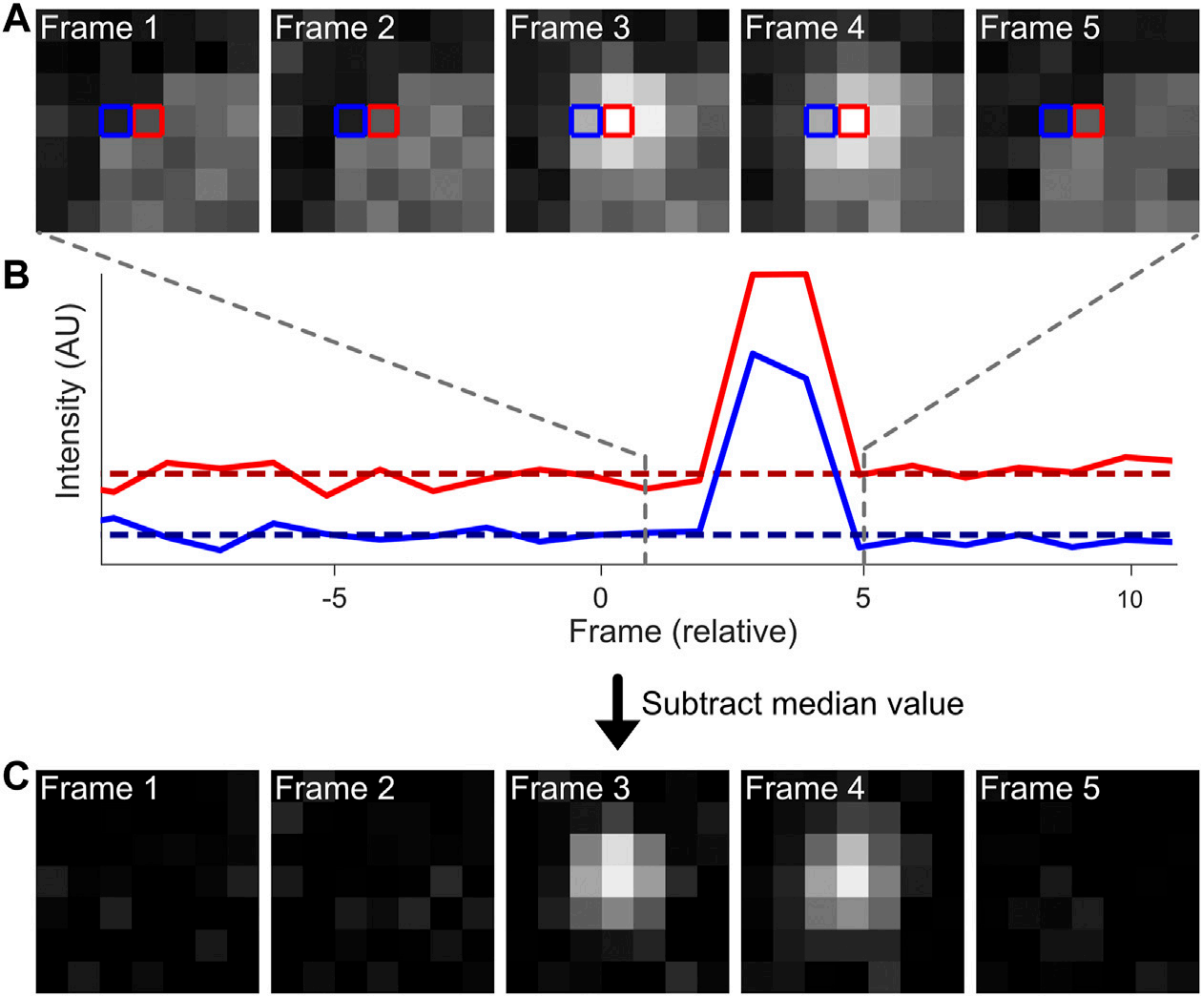
Computational workflow of a temporal median filter. Fluorescence emissions from individual, blinking fluorophores are often present on top of an inhomogeneous background **(A)**. The intensities of individual pixels can be analysed over a period of time [**(B)**, solid lines]. The median intensity value per pixel (dotted lines) can be extracted and subtracted from the original intensity data to adequately remove the background from the dataset **(C)**.

Raw single-molecule microscopy movie data often contains non-structured background noise with different photophysical characteristics as the fluorophores of interest, caused by for example residual out-of-focus fluorophores e.g. in the immersion oil or sample buffer, or by autofluorescence within the biological sample itself (Turkowyd et al., 2019, 2020). Additionally, out-of-focus fluorophores under HiLo or TIRF illumination will display different blinking characteristics compared to in-focus fluorophores, and can therefore also be filtered out. It can have a detrimental effect on localization efficiency (i.e. minimizing false positive and false negative localizations) and accuracy when identifying single-molecule emissions from the imaging data. The impact of background noise can be lowered by globally subtracting average background levels from the raw movie data. This, nevertheless, does not adequately capture temporal changes. Temporal median image filtering provides a solution to this problem (Figure 1).

Briefly, because *on/off*-switching of fluorophores in SMLM is equilibrated towards the *off*-state, the median intensity of a pixel is a good approximation of the background noise. Thus, the operating principle of temporal median image filtering is that for each pixel at time t, the median value of the pixel in the time interval t−i/2 to t + i/2 is computed and subtracted at time t (Figure 1B) (Hoogendoorn et al., 2014). The value i is user-defined, and should be substantially higher than the longest *on*-period of single emitters (at least twice; normally a value of ∼50 frames can be used), and is capped at high ends by unreasonable analysis times or temporal fluctuations in background intensity. A fast version of this algorithm is implemented as an ImageJ plugin (Jabermoradi et al., 2021). Temporal median image filtering should be avoided if the equilibrium of blinking is favored towards the *on-*state (i.e. > 50% of the time fluorescently active), since this would result in active removal of fluorescent signal rather than background. Additionally, this pre-processing step does not accurately reduce temporal heterogeneous background fluctuations (i.e. non-specific binding events).

Our code corresponding to module 1 can be found here: (https://colab.research.google.com/drive/1XKMP5BQWhUkQuKAjTkBgkaHy9bGjR23H or https://github.com/Endesfelder-Lab/SMLMComputational, also Supplementary Pseudocode S1). The required input is a raw SMLM movie, and the code module outputs a temporally-median-filtered movie with identical dimensions. Looping over every pixel in every frame, the pixel intensity values from i/2 before the current frame to i/2 after the current frame are extracted. Alternatively, if the frame is at the beginning of the movie, the pixel values are extracted from frame 0 onwards, and expanded further than i/2 after the current frame (and similar at the end of the movie). The median value is determined from this range, subtracted from the current pixel intensity, and stored in a new data array. These steps are then repeated for all pixels and all frames.

This concept can be taken one step further by first determining a localization and then calculating the local background from the spatiotemporal voxels in which no fluorescence of this emitter is present, followed by repeating the localization step. This has been realized by the SMALL-LABS software package (Isaacoff et al., 2019; Martens et al., 2021). Alternatively, temporal filtering can be based on minimum values to have a robust estimator at high fluorophore densities (Ma et al., 2021), or heterogeneous background can be assessed and restored via a neural network (Krull et al., 2019; Möckl et al., 2020). sCMOS-induced noise should be addressed separately (Diekmann et al., 2021; Zhang et al., 2021).

### Module 2: Localization

**FIGURE 2:**
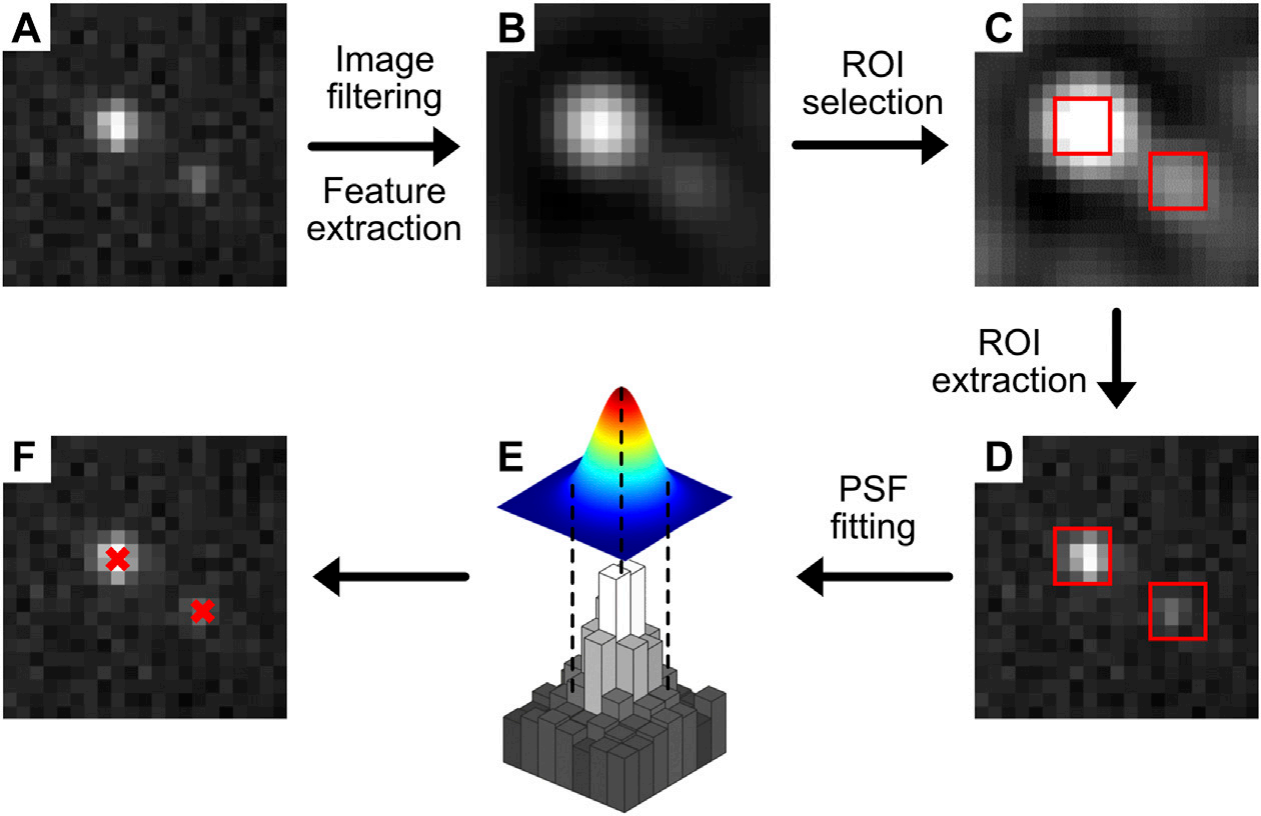
Typical localization methodology. A raw image **(A)** is filtered to enhance features that likely contain emitters **(B)**. From this filtered image, ROIs (red squares) are selected **(C)** and used to extract the PSF data from the original image **(D)**. This region is then fitted by a PSF model (e.g. commonly a 2-dimensional Gaussian) **(E)**, and the localizations with sub-pixel precision are displayed or used for further analysis **(F)**.

Determining the positions of individual fluorescent emitters to translate the SMLM movie data into SMLM localization data is the primary computational effort in SMLM imaging. Here, localization algorithms determine the sub-pixel accurate position of each point-spread function (PSF) of single fluorophores in the raw movie data (Figure 2). Principally, these localization routines consist of two steps, although methods are developed that merge these steps: 1) region-of-interest (ROI) selection, in which the presence or absence of a PSF is determined; and 2) sub-pixel localization of the emitter in the ROI. These steps are the basis of many user-friendly, open access software packages, such as ThunderSTORM, rapidSTORM, SMAP, Picasso, QuickPALM and GDSC SMLM (Henriques et al., 2010; Wolter et al., 2012; Ovesny et al., 2014; Schnitzbauer et al., 2017; Ries, 2020; Herbert, 2021).

Sub-pixel localization fitting procedures can benefit from fitting raw SMLM movie input, rather than a temporal-median-corrected movie (Module 1), if they e.g. take camera noise models into account that are effectively removed by temporal median image filtering. Thus, step 1 can be performed on the output of Module 1, while step 2 should be performed on ROIs extracted from the input of Module 1 (i.e. raw SMLM movie). However, in certain cases, such as when encountering hot pixels or patterned background fluorescence, the increased localization precision from running localization on raw movie data does not offset the removal of background.

The code belonging to this module can be found here: (https://colab.research.google.com/drive/1Jir3HxTZ-au8L56ZrNHGxfBD0XlDkOMl or https://github.com/Endesfelder-Lab/SMLMComputational, also Supplementary Pseudocode S2). A raw SMLM movie, or alternatively the output from Module 1 should be supplied as input, and a localization list with (frame*, x, y,* intensity) columns will be stored as output. Briefly, every frame in the temporal-median-corrected movie undergoes a difference-of-Gaussian (DoG) filtering to highlight PSFs. Local maximum positions are then found in the corresponding frame in the raw movie, which correspond to the approximate positions of PSFs. Looping over these local maxima, a small region of interest (7 × 7 pixels) is extracted, and the pSMLM code from (Martens et al., 2018) is used to extract the sub-pixel PSF position. This sub-pixel position is then added to the approximate PSF position, and added to the localization list.

Commonly, the ROI selection is performed *via* image filtering or feature enhancement. DoG filtering, like applied in this module’s code, is a common method used for edge detection (Marr et al., 1980). Alternatives to the DoG filter are the Laplacian of Gaussians [LoG; (Tinevez et al., 2017)] or a β-spline wavelet filter (Izeddin et al., 2012a).

Sub-pixel localization has seen many improvements in the past decades and several localization software challenges benchmarked different algorithms for different data scenarios (Sage et al., 2019). Because a 2-dimensional Gaussian function is a good approximation for the PSF of in-focus fluorophores, iterative algorithms based on fitting a Gaussian function are often used (Mortensen et al., 2010; Stallinga and Rieger, 2010), providing good accuracy especially when using a maximum likelihood estimator (MLE) fitting procedure (Smith et al., 2010). Possible fast analysis methods are centroid-based (Cheezum et al., 2001), phasor-based (Martens et al., 2018, 2021), which is used here because of the low computation time and good accuracy, or radial-symmetry-based (Parthasarathy, 2012). Another type of algorithms that more accurately simulate and localize PSFs also exists, based on theoretical or measured optical wavefronts (Liu et al., 2013; Shechtman et al., 2014; Aristov et al., 2018; Xu et al., 2020) or on measured PSFs (Babcock and Zhuang, 2017; Li et al., 2018). Recently, deep-learning-based methods combine the ROI selection and sub-pixel localization with excellent results (Nehme et al., 2020; Speiser et al., 2021).

The sub-pixel localization step can additionally be used to obtain information about the 3-dimensional position of individual emitters. This requires additional optical elements in the microscope’s emission path such as elliptical lenses (Huang et al., 2008), deformable mirrors (Izeddin et al., 2012b; Martens et al., 2021), or custom phase masks (Shechtman et al., 2014), or is based on simultaneously imaging slices at different depths (Juette et al., 2008; Louis et al., 2020). In all cases, information about the *z-*position is encoded in the shape of the PSF, and thus more complex localization analysis needs to be performed (Aristov et al., 2018; Li et al., 2018).

Importantly, it is assumed that every ROI only contains a single fluorophore for most of these implementations. This is not always the case, especially in high-density samples. While the common approach in the SMLM community is to prevent these high densities experimentally, there are computational approaches designed specifically for high density and multi-emitter fitting (Holden et al., 2011; Zhu et al., 2012; Marsh et al., 2018; Nehme et al., 2020; Speiser et al., 2021). Additionally, the localization result should be checked for inhomogeneous distribution artefacts (e.g. bias towards the center of a camera pixel), which can be especially important when the experiment requires quantification of repeating patterns.

### Module 3: Localization Merging

The movie acquisition speed (i.e. time per imaging frame) during SMLM imaging has to be optimized based on the method (i.e. STORM, PALM, etc.,) and dependent on technical and biological sample factors, e.g. whether a static or dynamic sample is imaged (i.e. super-resolution imaging or single-particle tracking). During imaging of static samples, fluorophores switched to their *on-*state normally remain in this state for ∼10–50 ms (STORM) or ∼100–500 ms (PAINT), depending on the experimental set-up. Additionally, the fluorophores can go in various temporary dark-states (blinking), meaning that no emission can be detected for several frames (Dickson et al., 1997; van de Linde and Sauer, 2014; Berardozzi et al., 2016). Because SMLM acquisition speed is static, it is likely that a single fluorophore can be in its *on*-state for more than one imaging frame. This means that the same fluorescent event is recorded multiple times over consecutive frames, but could be “skipping” one or multiple frames due to fluorophore blinking.

**FIGURE 3:**
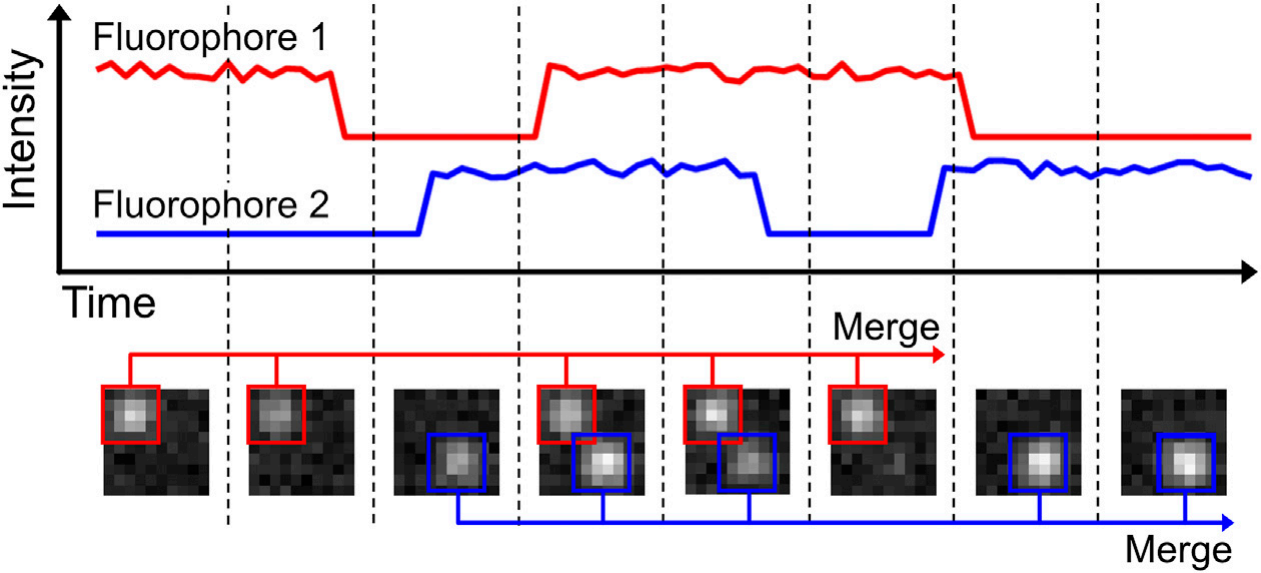
Localization merging. In the shown example, two fluorophores are present. Both fluorophores are emitting for multiple frames, but are also blinking during this period. The localization merging routine identifies emitters that emit over multiple frames, accounting for possible blinking periods. Merging the individual localizations into one increases accuracy of fluorophore quantification and emitter localization precision.

It can be advantageous to merge these multiple recordings of a single emission down to a single event (Figure 3). First, this will provide a more quantitative overview of the sample, which can help with e.g. counting of fluorophores. Secondly, merging multiple events allows for *de facto* higher photon levels (N) per localization, which scales with localization precision by 1/√N (Rieger and Stallinga, 2014). Merging is readily available in the post-processing of many SMLM software packages, such as ThunderSTORM, SMAP and Rapidstorm (Wolter et al., 2012; Ovesny et al., 2014; Ries, 2020). In Rapidstorm, the merging is implemented as a Kalman-filter, which improves the merging quality. Care should be taken when performing localization merging on high-density datasets, as this could result in linking different fluorophores to each other, rather than linking multiple emissions from a single fluorophore.

Our code belonging to this module can be found here: (https://colab.research.google.com/drive/16ooyjTonAP3xvsQKCv_uxWcUp1hB8msC or https://github.com/Endesfelder-Lab/SMLMComputational, also Supplementary Pseudocode S3). It requires a localization list (at least containing frame, *x*, *y* position) as input, and stores a corrected localization list as output. The code itself loops over all localizations on a given frame. For every localization, it is checked whether there are localizations in the next 1 or 2 frames that are closer than a user-defined maximum distance. This pair of localizations is then given an identical “trajectory-id”. After looping over all localizations, the localizations that have the same trajectory-id and do not belong to special cases [e.g., on purpose placed fiducial markers for drift correction (see Module 4a)] are merged. This is performed by taking their collective, intensity-weighted mean position, minimum frame value, and summed intensity. The original localizations are then replaced by this merged localization.

Localization merging can be reasonably expanded to work on the level of the movie data. This would involve re-performing the localization module on the summed raw data of merging events.

### Module 4: Drift Correction

SMLM data is recorded in movies (and not in single image snapshots), and thus the data is acquired over substantial time periods, typically in the order of tens of minutes. The obtained localization precision in the final reconstructed image, that summarizes all localizations from all imaging frames, is in the order of nanometers. But high image resolutions can only be achieved and the results are only interpretable if some technical criteria are fulfilled, e.g. sufficient fluorophore labeling density and detection efficiency as well as an absence of temporal drift during the movie acquisition (Vojnovic and Endesfelder, 2020). For the latter, it thus is important that the sample itself moves only very minimally with respect to the detector throughout the acquisition. However, this is challenging, if not impossible, to achieve via merely stabilizing hardware (even if the setup has good heat dissipation and a vibration-damping module). Therefore, additional drift correction procedures are used, either on-line (directly during acquisition) or off-line (post-processing and correcting the localization data after acquisition).

A distinction should be made between axial (i.e. in the *z*-direction) and lateral (i.e. in the *xy-*direction) drift for two reasons. First, axial drift is much more detrimental to the acquisition, because the emitters are only in focus in an axial slice of about 600 nm (Franke et al., 2017). Second, many microscopes have axial stages equipped with piezo-stages that provide accurate and repeatable precision of ∼1–5 nm, while the lateral stage is usually not equipped with a piezo-stage, which limits on-line correction of lateral drift to a micrometer-accuracy. For these two reasons, axial drift correction is often performed on-line *via* a hardware add-on based on the internal reflection of an (infra) red laser (Liron et al., 2006), while lateral drift correction is performed off-line using one of the numerous variations of the methodology that we outline below. Briefly, fiducial marker drift correction (Module 4a) can be used for any sample, but requires introduction of steady-fluorescent markers in the sample, while cross-correlation drift correction (Module 4b) calculates and corrects for drift directly from the samples’ features, but requires static data and thus cannot be used for highly dynamic samples or particle tracking studies.

### Module 4a: Drift Correction by Fiducial Markers

**FIGURE 4:**
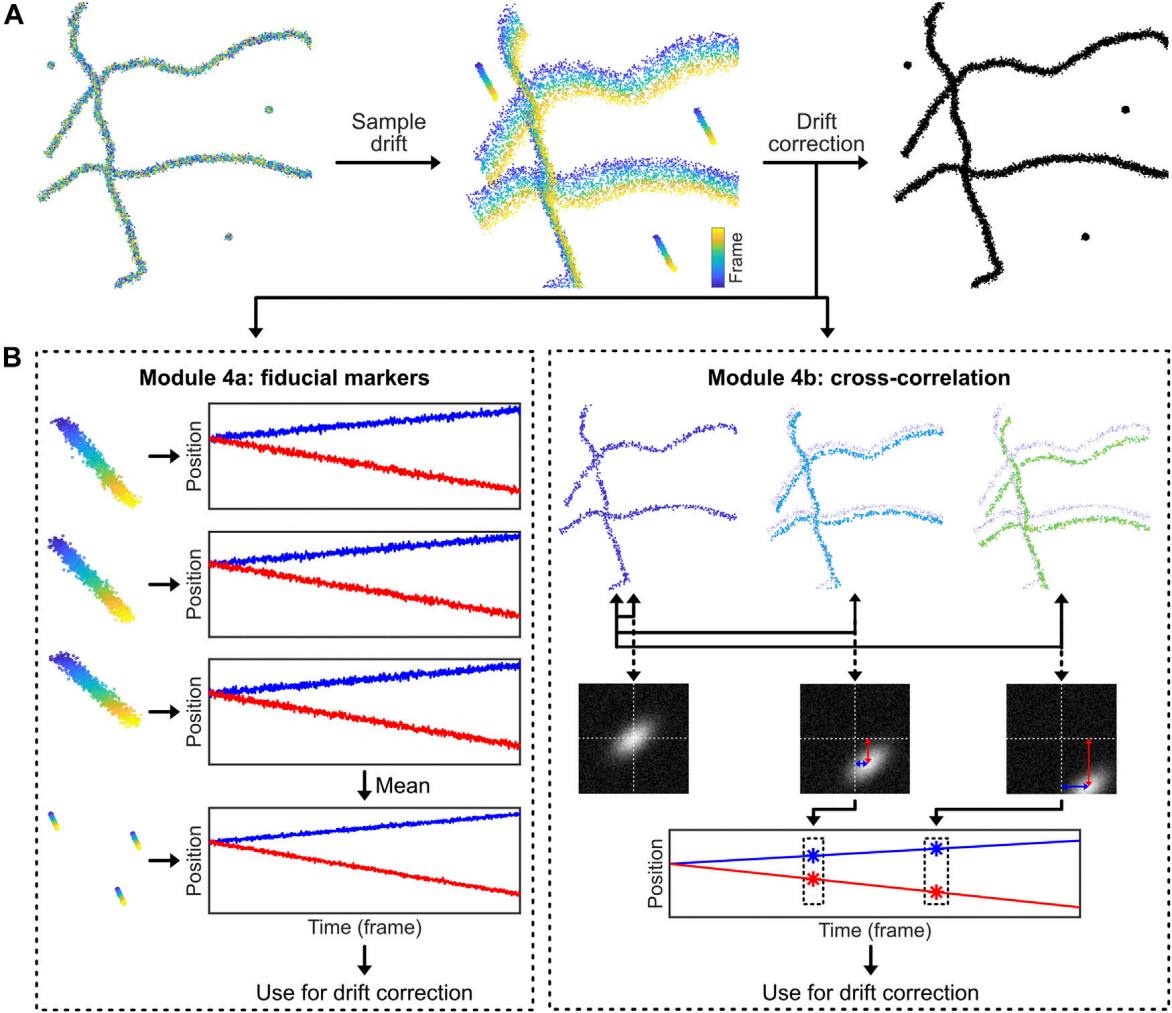
Drift correction methodologies. Most SMLM experiments exhibit sample drift, so the localizations obtained from each fluorophore depend on their detection time within the SMLM acquisition. The reconstructed SMLM image, which combines all localizations from the SMLM experiment, is therefore affected by the overall drift, which directly affects the structural resolution and as such can introduce misleading artifacts (e.g. smearing clusters to filaments). Drift correction methods correct this. **(A)**: Drift can be corrected by introducing fiducial markers that can be tracked with high precision. **(B)**: Alternatively, the displacement of the biological structure at different time points can be analysed *via* temporally cross-correlating the data.

A conceptually simple way to measure and correct sample drift is to introduce stable fluorescent fiducial markers (Balinovic et al., 2019). These markers are bright, non-blinking emitters [often nanoparticles that have many individual fluorophores bound to a support structure such as polystyrene beads, possibly excited away from their absorption maximum (Balinovic et al., 2019)] that emit stable and bright fluorescence throughout the acquisition time. By incorporating and tracing the signal of multiple fiducial markers in every field of view (FoV) that is recorded, the drift of the sample can be assessed (Figure 4). The displacement of this drift trace from its original position at time point zero can then be subtracted from all localization data, allowing for effective drift suppression that in practice achieves a precision of about 3–5 nm (Balinovic et al., 2019). In case the fluorescence of the fiducial markers is not stable, if the fiducial markers cannot be distinguished from sample signal, or if the marker moves separately from the sample, this method will provide inaccurate results. Moreover, with high intensity fiducial markers, camera oversaturation will result in bad fitting, causing artefacts. Finally, if a marker with multiple fluorophores is used, bleaching of a single fluorophore can bias localization, if only a small number of fluorophores are remaining on the marker (Balinovic et al., 2019).

The code belonging to this module can be found here: (https://colab.research.google.com/drive/1U-yiO56r4uG92hnq1KKAKAjy4IeW8n_I or https://github.com/Endesfelder-Lab/SMLMComputational, also Supplementary Pseudocode S4). For fiducial marker-based drift correction, the fiducial localizations must be selected and isolated. In our module, we identify the fiducials by their constant signal: appropriately chosen fiducial markers will be present throughout the entire movie acquisition. Alternatively, the fiducial markers can be isolated based on higher fluorescence intensity compared to dSTORM, PALM, or PAINT fluorophores, or selected based on their position by hand (i.e. based on coordinates). After performing a single-particle tracking routine (see Module 3), the position of each marker is compared to the position of the same marker in the first frame of the movie. This yields time traces of the drift for every single fiducial marker that have a temporal resolution of one frame. These time traces of individual fiducial markers are averaged to obtain a single drift trace. This averaged drift trace usually has better accuracy than the individual traces because inaccuracies in localization are averaged out. The drift trace is then subtracted from all localizations in the whole dataset, which effectively removes the effects of sample drift. Fiducial marker drift correction is normally applied to 2-dimensional data only, but can easily be expanded to include axial drift, assuming that a 3-dimensional localization procedure is used.

### Module 4b: Drift Correction by Cross-Correlation Methods

Data belonging to structural samples that do not change themselves during the acquisition (i.e. SMLM images of immobilized, non-dynamic samples as being obtained by dSTORM or PAINT imaging), can effectively be drift-corrected by visualising the data at different time points and comparing these visualisations (Mlodzianoski et al., 2011) (Figure 4). In principle, drift correction by cross-correlation methods is based on the fact that the image generated by the localizations is identical throughout the acquisition time. This means that e.g. for a dataset comprised of 1,000 frames, a visualisation of the structure can be generated from imaging frames 1–100, which can be compared with a visualisation generated from imaging frames 101–200, etc. If drift is present, the second visualisation will be offset from the first. Measuring this offset over time using consecutive data subsets, the overall drift trace can be obtained and corrected for. Drift correction via cross-correlation requires stable, unmoving datasets. In case the structure itself is flexible or moves throughout the data acquisition, this method will silently fail. In addition, heterogeneous sample drift or sample rotation (i.e. caused by uneven matrix contraction) should be prevented.

In our module 4b found here: (https://colab.research.google.com/drive/1DUhUxeCnYXxD7ZkL9NcIDxE6VV7fnzvQ or https://github.com/Endesfelder-Lab/SMLMComputational, also Supplementary Pseudocode S4), we therefore generate multiple images from different time bins. We then calculate cross-correlations between the visualisations at each time bin and the visualisation at the start of the SMLM acquisition (= first time bin). The spatial position of the intensity maximum of each cross-correlation provides a good measure for the drift. This position is identified and attributed to the temporal centre of each bin. The drift trace is based on these points, and—in our module as well as for most cross correlation implementations—non-linearly interpolated to smooth the trajectory. The drift trace is subtracted from the original localizations. This can additionally be expanded to three-dimensional data by taking *z*-slices, and comparing those similarly.

This technique can be expanded to redundant cross-correlation (RCC) (Wang et al., 2014), in which the temporal bins are not only compared to the first, but to all bins. This increases computational effort, but results in higher accuracy. Alternatively, the positions of the emitters at different time points can be compared with each other. The mean shift of the localizations over time is a measure for the drift, similar to the shift of the maximum position of the cross-correlation images, (Cnossen et al., 2021; Fazekas et al., 2021).

### Module 5: Chromatic Aberration Correction

**FIGURE 5:**
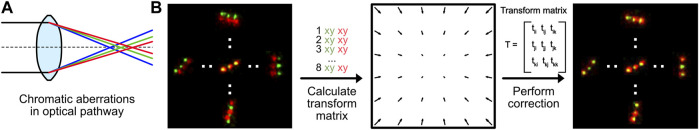
Chromatic aberration correction. (A) Optical elements in any microscope will refract light of different wavelengths differently, causing chromatic aberrations. **(B)** Chromatic aberrations can be corrected by calculating a transformation matrix based on pairwise positions of fluorescent markers measured at different wavelengths. This transformation can be used to correct the instrument-specific chromatic aberration.

All optical components in a microscope experience chromatic aberrations: light is refracted slightly differently based on its wavelength (Figure 5A) (Erdelyi et al., 2013). Today, almost every optical element in a fluorescence microscope is corrected for chromatic aberrations. Thus, standard diffraction-limited fluorescence microscopy can be performed without further chromatic aberration corrections. Nevertheless, even for high-quality optics, a residual chromatic shift in the nanometer range shift remains. This is enough to hamper multicolor super-resolution imaging and creates a mismatch of images generated by fluorophores with different emission wavelengths (Zessin et al., 2013) (Figure 5B, left). This chromatic aberration is microscope-specific and directly dependent on the optical path and individual components. It thus has to be measured for each setup individually. Nevertheless, it is a static shift (as long as no components change), so it does not need to be repeatedly measured for every new experiment.

In our module 5 (https://colab.research.google.com/drive/1UH0BIuHUJFjF_hXtO3rwdOLTl45LkzLz or https://github.com/Endesfelder-Lab/SMLMComputational, also Supplementary Pseudocode S5), we correct the chromatic aberration by comparing data with identical ground-truth positions emitted at two different wavelengths. For the data-pairs, an affine 2-dimensional transform matrix is estimated. For microscopes with more than two color channels, such a matrix has to be estimated for all channels in relation to one reference channel. These transformation matrices can then be used to correct the chromatic aberration from all datasets measured with the same microscope and color channels.

As mentioned, a requirement for chromatic aberration correction is a sample that is identical for multiple emission wavelengths. In this module, we have used a so-called “DNA-PAINT nanoruler” which has identical “docking positions” for both, ATTO542 and ATTO655 fluorophore DNA oligos used as reporters (emission peaks at 561 nm (“green”) and 680 nm (“red”), respectively). Those reporters can repeatedly bind and unbind during the SMLM acquisition. The chromatic aberrations in our microscope results in green positions that are localized slightly further to the outside of the image than the red positions (Figure 5B, left). We then hand-picked green and red DNA-PAINT position pairs, and used their relative positional shifts to calculate the affine transformation matrix (Figure 5B, middle). This transformation is then applied on either the image created from the red localizations, or directly on the red localizations. This effectively reduces the experienced chromatic aberration (Figure 5B, right). We note that this analysis method can also be applied for translational offsets, introduced in e.g. dual-view camera systems.

### Module 6: Image Generation

**FIGURE 6:**
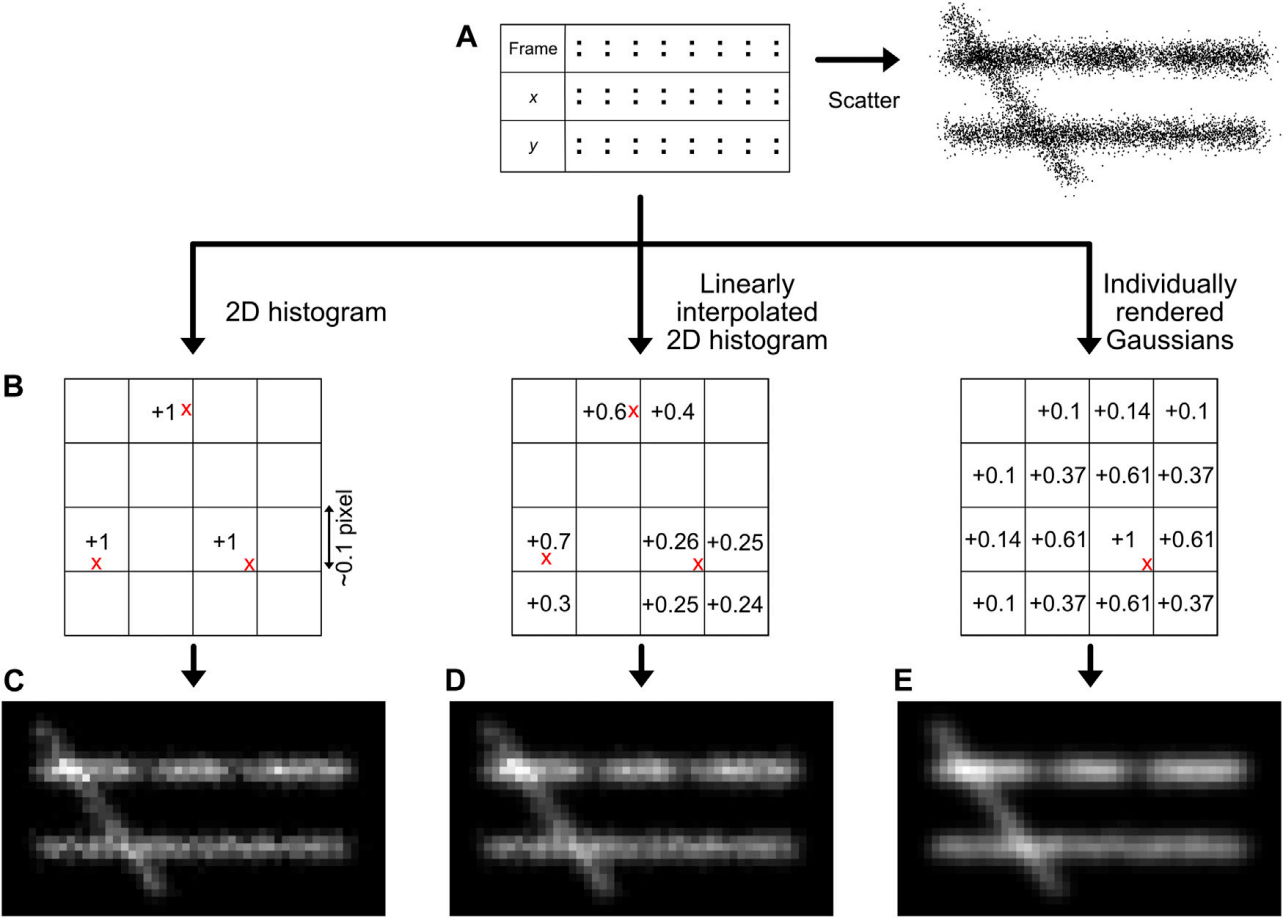
Image generation methods. The simplest way for image generation is a scatter plot, although this does not reflect the localization density accurately **(A)**. The localizations (red crosses) can also be placed on a sub-pixel grid **(B)**, and the bin intensities can be increased based on the position of the localizations. The methods described in this manuscript are the creation of a 2D histogram **(C)**, a linearly interpolated 2D histogram **(D)**, or individually rendered Gaussians **(E)**. Note the regions with lower density (66% lower density compared to the surrounding) and the different intensity between the two horizontal lines (30% lower density) that are more clearly interpreted by histogram images compared to a scatter visualisation.

A main goal of SMLM, but especially of structural super-resolution imaging, is the generation of super-resolved images from the localization data. However, this is not as straightforward as it may sound, since ultimately the dataset of SMLM localizations are essentially 0-dimensional points. While plotting these localizations as a scatter plot may provide some information (Rust et al., 2006), the symbol size and shape can be arbitrary and the scatter plot does not adequately visualise local emitter density (Figure 6A). Thus, it should normally not be used. Generally, it must be noted that all forms of image visualisation decrease the resolution (as every pixelation variant, even when adjusted to the experimental localization precision of the data, ultimately sorts the data in bins), but are often very useful for human interpretation. It is therefore recommended that downstream quantitative efforts are focused on the localization list rather than generated images.

One could reason that each localization can be visualised as the central point of a 2-dimensional Gaussian function with a full-width half maximum determined by the localization precision. This procedure would be conceptually very similar to the physical representation of regular brightfield microscopy, which can be interpreted as many simultaneous localizations generating PSFs with a width determined by the optical resolution. However, this methodology actually results in a loss of visual resolution, as it effectively blurs the original structure by the visualization method in addition to blurring caused by the localization error, resulting in a √2 resolution loss (Baddeley et al., 2010). Moreover, the rendering of several thousands to millions of 2-dimensional Gaussian functions is computationally expensive and thus unrealistic *via* a computation processing unit (CPU) only (and instead requires e.g. using a graphical processing unit (GPU) that works in a highly parallelized and optimized manner), unless well-optimized code and functions are used (Ries, 2020).

A quantitatively better way for localization visualisation is to place the localizations in user-defined sub-pixel bins, normally ∼10–15 sub-bins per original imaging pixel in each dimension (Figure 6C) (Nieuwenhuizen et al., 2013). It is important to choose this sub-bin value cautiously, as the super-resolution image pixel size should be in the range of the localization precision (Nyquist-Shannon sampling theorem) (Nyquist, 1928; Shannon, 1949), also see Module 9 to determine the localization precision. If a smaller sub-bin value is chosen, it could lead to visualisation of non-existing details, hindering correct interpretation. This can additionally be subject to a pseudo-Gaussian kernel to spread the intensity to surrounding pixels, which is especially valuable on datasets with sparse signals. This is the approach taken in our module, but is also used standard in e.g. the ThunderSTORM software (Ovesny et al., 2014).

A more sophisticated method, also shown in our module and first published with the software Rapidstorm, is to linearly interpolate the localizations on a sub-pixel raster (Figure 6D) (Wolter et al., 2010). In the basis, this method is similar to localization binning in sub-pixel bins, but additionally, neighbouring pixels are also populated based on the distance from the localization to the center of the main sub-pixel bin, preventing discretization errors.

Our module (https://colab.research.google.com/drive/14OCvRUAUFp9JXK6HVyj18fndGY92-Dsx or https://github.com/Endesfelder-Lab/SMLMComputational, also Supplementary Pseudocode S6) implements all three methods. Regular 2-dimensional histogram visualisation is straightforward in Python and MATLAB, since this is a built-in function in both languages. For linearly interpolated histograms, for every localization, the correct sub-pixel bin is found, as well as the distance to the center of the sub-pixel bin. This distance in *x* and *y* is used to calculate the relative intensity in the neighbouring pixels. Image generation based on individual Gaussian reconstruction is done by looping over every emitter and over every sub-pixel in the reconstructed image, and increasing this value based on the distance to the emitter position.

More involved methods are investigated by Baddeley et al. (2010), and show that adaptive quad-tree histograms and visualisation based on Delaunay triangulation have distinct advantages for SMLM image generation, at the cost of computational complexity.

### Module 7: Single-Particle Tracking (spt)

In contrast to structural SMLM imaging, spt is a methodology in which moving fluorescently-labeled objects are tracked over time. Rather than generating an image, assessing and interpreting this movement is the goal of spt. Computational efforts are therefore fundamentally different in spt from those in structural imaging (Chenouard et al., 2014). Analysis consists of three main computational efforts: 1) localizing moving PSFs, 2) linking the localizations of single particles from consecutive imaging frames into trajectories and 3) analysis of the dynamics and diffusional states of the particles from their trajectories.

### Localization

Localization efforts required in spt are largely similar to localization efforts required in structural SMLM (Module 2). However, the inherent movement of fluorophores in spt causes deviations of the measured PSF from a theoretical PSF. Software explicitly designed to localize static PSFs can therefore fail when localizing moving PSFs. Downstream processing of spt also dictates that there is a higher priority on detecting the fluorescent emissions than there is on localization precision: because statistics from the fluorophore trajectories are averaged over many linkages, this effectively lessens the influence of localization errors. This results in localization efforts designed for spt to be robust (i.e. high accuracy on fluorophore detection) rather than precise, e.g. as implemented in Trackmate (Tinevez et al., 2017).

### Linking of single Fluorescent Emissions Into Particle Trajectories

Linking single fluorescent emissions into particle trajectories is a conceptually simple problem: localizations in subsequent frames possibly belong to the same emitter, and these should be linked together to obtain a trajectory through time, which can be further analysed. In its easiest form, tracking can be performed by determining the nearest localization in the next frame for each localization. Then, as long as the jump distance (JD) between these localizations is lower than a user-defined value, the localizations are linked together and form a track. This methodology is commonly known as nearest-neighbour tracking.

However, nearest-neighbour tracking is not a final method due to several reasons. First, there could be several localizations within the search radius and the closer one could simply be the wrong choice (i.e. two trajectories are crossing each other, or localizations are found due to autofluorescence). Second, fluorophores can blink for one or multiple frames, which effectively means that a “gap” can be present in the trajectory, which should be accounted for. Third, since Brownian diffusion results in a noncentral chi (Rayleigh) distribution of jump distances, there is no well-defined maximum jump distance. Fourth, a population can consist of more than one diffusive state, meaning that the user-chosen maximum jump distance is even less well-defined. Last, nearest-neighbour tracking is prone to introduce artifacts as there is no way to end a trajectory if any localization is present within the defined radius. This will introduce false linkages within the trajectories (e.g. caused by autofluorescence or another molecule appearing in close proximity, e.g. in molecular clusters). These artifacts can be lessened by reducing the search radius, but this will lead to many truncated trajectories (see third and fourth argument). All that being said, nearest neighbor tracking in low density and low noise datasets will experience a neglectable effect from all these criticisms. Meanwhile, it does not introduce any algorithmic bias which easily happens the more *a priori* knowledge and assumptions are taken into account using more advanced methods.

Still, solutions for more dense or background-intense spt are a field of on-going method development (Chenouard et al., 2014). All of those algorithms incorporate *a priori* knowledge. E.g. the Icy software (de Chaumont et al., 2012) uses a Bayesian model with multiple hypothesis tracking (MHT) that yields more accurate results especially for weak fluorescent signals (Chenouard et al., 2013). Or alternatively, in TrackMate (Tinevez et al., 2017), tracking is formulated as a linear assignment problem (LAP) (Jaqaman et al., 2008), in which a computational cost factor balances localization-to-localization linkages and track initialisation and termination (i.e. minimizing wrong linkages). Also, localization and tracking steps can be combined, e.g. alternatingly performing localization and tracking to verify each other, as implemented in multiple-target tracking (MTT) (Sergé et al., 2008).

### Quantitative Analysis of Trajectories

**FIGURE 7:**
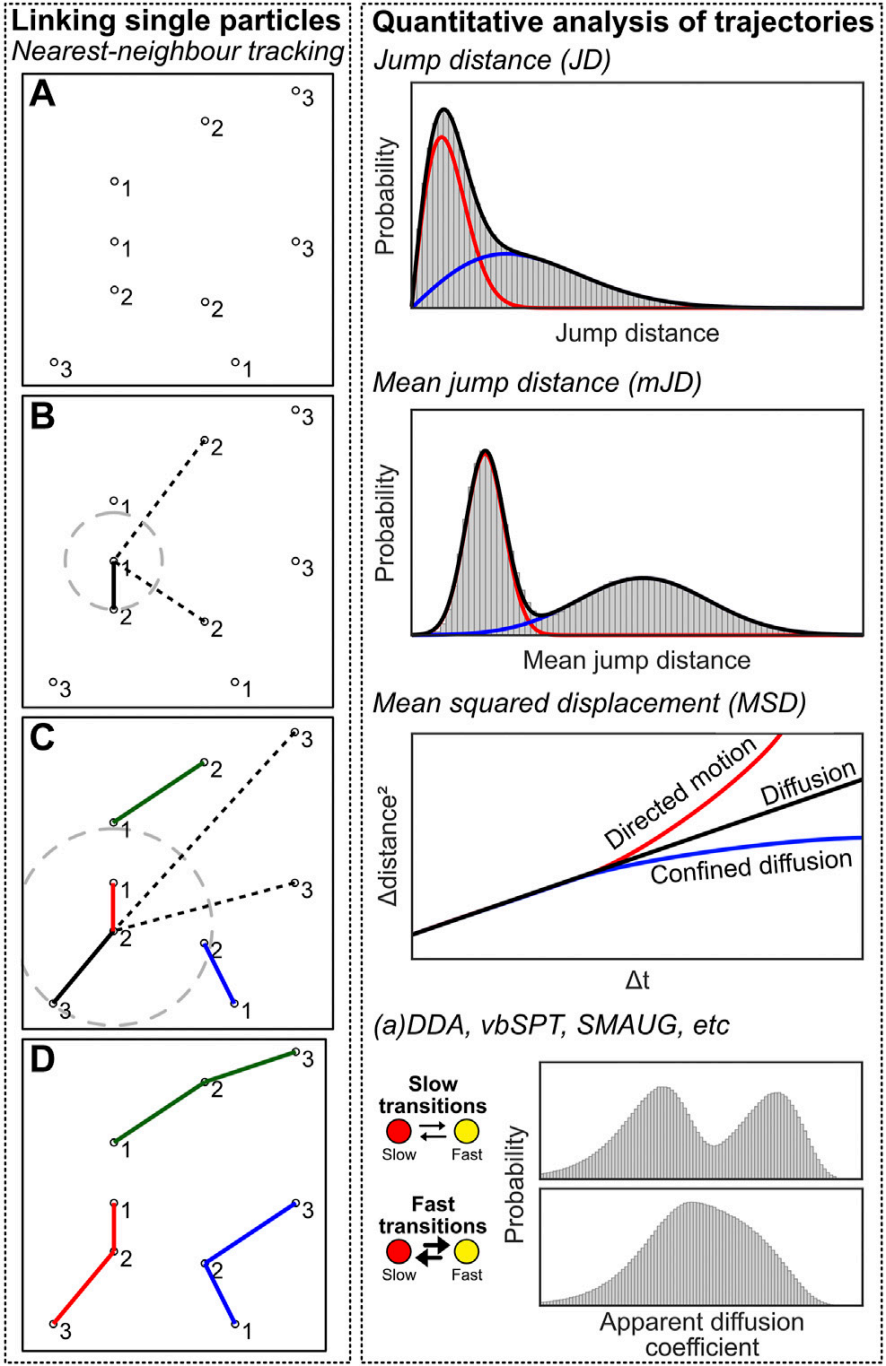
Single-particle tracking computational approaches. Left: Individual emitters have to be linked to create trajectories. Individual emitters have been localized at multiple frames **(A)**, and the nearest neighbour localization in frame n+1 is determined for every localization at frame n, and a linkage is created between these localizations **(B,C)**. This creates trajectories that can be further analysed **(D)**. Right: quantification analysis of trajectory data. See the main text for details on the methods.

After the localizations are linked into trajectories, the underlying dynamics can be analysed to interpret the data (Figure 7, right). The simplest method is to create a JD histogram, and fitting this histogram with one or multiple diffusive populations from which apparent diffusion coefficients (D*) can be extracted (Schütz et al., 1997; Vrljic et al., 2002). However, analysis of a JD histogram does not have sufficient resolving power if two or more populations with small differences are present; in this case, the mean jump distance (mJD) of every trajectory can be determined, which provides stronger differences, i.e. separated maxima, between populations (Turkowyd et al., 2019; Martens et al., 2020). An analytical correct solution of mJD histograms is impossible, as the underlying data has different statistical origins due to varying trajectory length. However, for sufficiently long trajectories, mJD values will approach, and thus can be well-approximated by, a Gaussian (central limit theorem) from which the diffusion coefficient can be extracted. In our module, we implemented both analyses.

In our module (https://colab.research.google.com/drive/1v4N6os8cdHqilDLguYUGrKmlRM8vcG_8 or https://github.com/Endesfelder-Lab/SMLMComputational, also Supplementary Pseudocode S7), we implemented nearest-neighbour tracking while taking blinking into account. This is identical code to the tracking performed in Module 3, albeit with a larger maximum jump distance. Next, the script loops over every trajectory, and consecutively over every localization in the trajectory, except for the last one. The Euclidean distance between this localization and the next localization is calculated and stored if there is 1 frame temporal distance between these localizations. Localizations that do not have a jump distance calculated like this get a value of −1 to easily filter out in later steps. Next, the mean jump distance for the complete trajectory is calculated for every trajectory. The jump distances or mean jump distances are then extracted and plotted in a histogram, after which a non-central chi distribution (JD) or Gaussian approximation (mJD) is fitted to the histogram. This analysis routine is performed on free diffusion of two populations of beads with different sizes, and we show that fitting the data with a single population does not provide satisfactory results. Please also note that the number of bins in the histogram can have an effect on the fitting procedure, and care should be taken to assure that fitting is robust with respect to the bin size.

Another popular method involves calculating the mean squared displacement (MSD) of the trajectories, by taking the squared displacements over time (at Δt = 1, 2… *n*-1 for a trajectory with length *n*), averaged over all possible starting positions of the trajectory per *Δt* (Qian et al., 1991). These displacements are then plotted as a function of *Δt*, and yield 1) the diffusion coefficient D by the MSD curve slope; 2) the localization uncertainty by the intersection with the *y*-axis; and 3) the type of diffusion (i.e. pure diffusion, confined diffusion, directed motion, relatively) by the shape of the curve (i.e. linear, curved downwards, curved upwards) (Lee et al., 2017). However, the MSD is sensitive to noise in the case of short trajectories commonly obtained *via* sptPALM.

So far, these analysis methods assume that the diffusive state of the underlying trajectory does not change. However, this is commonly not the case in biological situations, e.g. in the case of DNA-binding proteins, where the proteins can be diffusing or be stably bound to the DNA. There are several software packages available that quantify transient states and their state-changing kinetics: (a)DDA [(analytical) diffusion distribution analysis] allows for analysis with a temporal resolution faster than the frametime (Martens et al., 2019; Vink et al., 2020a, 2020b), while vbSPT (Persson et al., 2013) and SMAUG (Karslake et al., 2021) specifically assume state-changing slower or on the same timescale as the frametime.

### Module 8: Clustering

By cluster analysis methods, localizations are grouped into coherent structures which helps to visualize and interpret structural data. There are several clustering approaches which can be categorized by their clustering model, e.g. connectivity-based (hierarchical), centroid-based, distribution-based, or density-based methods. Generally, clustering algorithms can be extended to colocalization algorithms when taking a second color channel in consideration (Malkusch et al., 2012; Rossy et al., 2014).

A simple approach is Ripley’s K-functions and its normalized variants (i.e. L- and H-functions), which measure the data density as a function of radius around every point in the dataset and compares it to random spatial distribution at same density (Ripley, 1977; Owen et al., 2010; Endesfelder et al., 2011). It does not require initial parameters but can only reveal whether clusters are formed and does not report cluster size accurately (Malkusch et al., 2013). Since the Ripley’s functions provide a value that is non-straightforward to interpret, it is normally compared against a differing biological condition (Rossy et al., 2013).

A common clustering algorithm that defines individual clusters is the K-means algorithm (Hartigan and Wong, 1979). It is a centroid-based and unsupervised method that finds the centroids of clusters by minimizing the summed distance of all localizations to the nearest clusters’ centroids. However, this approach requires the user to pre-determine how many clusters are expected, and is based on a spherical cluster model without considering noise.

**FIGURE 8:**
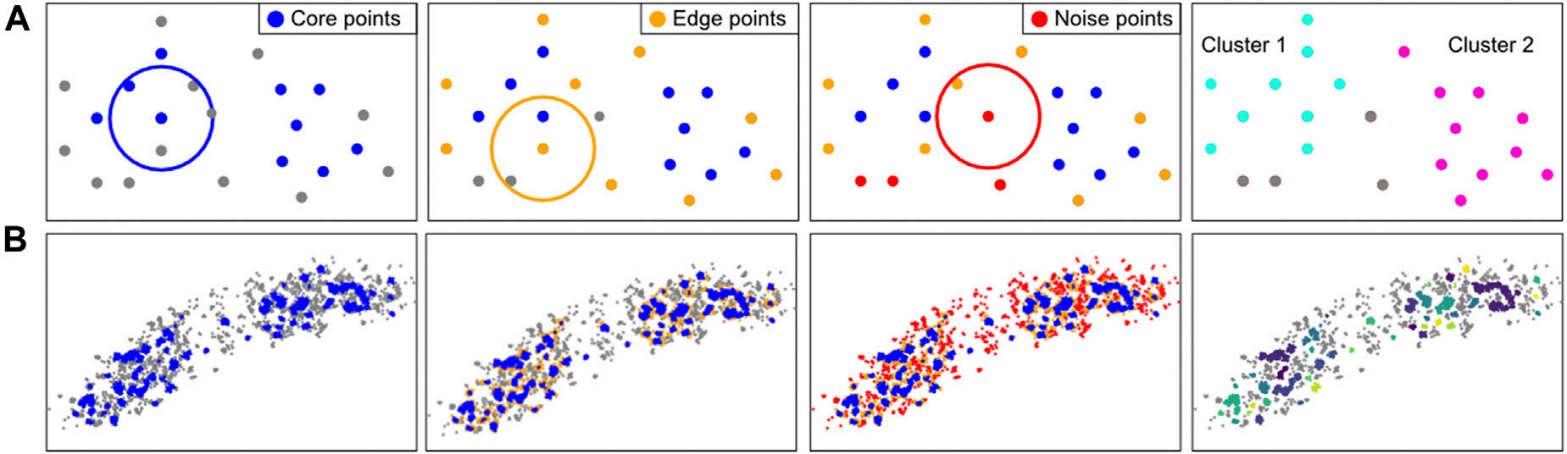
Computational approach of the clustering method DBSCAN. **(A)**: DBSCAN explanation. For every localization in a given FoV, the number of neighbours in a user-defined radius are counted. If there are more neighboring points than a (by the user) set minimal number of points (left), these points are considered core points (blue). All localizations that are not core points, but have a neighbour that is a core point, are considered an edge point (orange, second panel). Localizations that have neither enough neighbours nor proximal core localizations are considered noise points (red, third panel). The core and edge localizations together are considered to form a cluster. The identified clusters can be visualised and characterized (right). In this example, at least 3 neighbours are required for a localization to be considered a core point in the radius indicated by the circle. **(B)**: Application of the DBSCAN steps on an *E. coli* cell with fluorescently-labeled RNA polymerase.

Density-based algorithms can account for irregular shapes and noise. The most known algorithm of this kind in SMLM analyses is DBSCAN (Density-based spatial clustering of applications with noise), which is used in Module 8 (Figure 8) (Ester et al., 1996; Endesfelder et al., 2013). DBSCAN requires two parameters: 1) the radius in which adjacent localizations are considered as neighbours, and 2) the minimum number of points in this neighborhood required to initiate the cluster formation. Based on these criteria each point is labeled as a “core point”, “edge point” or “noise point”. Core and edge points belong to clusters, while noise points do not. This classification is then used to uniquely define the individual clusters.

Note that clustering methods require care (Khater et al., 2020), as all clustering algorithms tend to quantify clusters, even if these do not exist in the dataset, i.e. most methods lack a quality control and fail silently. Moreover, if blinking is not adequately corrected for (Module 2), this could influence clustering results. Next, non-spatially resolved clustering methods (i.e. Ripley’s functions) can be influenced by edge effects, e.g. where a uniform distribution inside a single cell can be quantified as non-uniform, because higher localization density inside the cell is contrasting with lower density outside the cell. Finally, DBSCAN could provide quantitatively poor results when directional heterogeneity exists on a same scale as the search radius.

Our module (https://colab.research.google.com/drive/1ruLv02SWFtlEAlZTkWSgHnucoGAZPDcF or https://github.com/Endesfelder-Lab/SMLMComputational, also Supplementary Pseudocode S8) implements DBSCAN. The script loops over all localizations, and first finds, counts, and stores the neighbouring localizations (over all frames) in a table. Afterwards, the core and cluster localizations are found based on the procedure described earlier. Finally, a recursion algorithm is employed to determine the individual clusters. Briefly, a loop is started over all core or cluster points, which is set to the current label-id. Next, the same loop is started over all neighbouring core and cluster points, if the original point was a core point. Only if no more neighbours without an assigned label-id can be found, the label-id is increased.

DBSCAN is widely used in SMLM analysis (Endesfelder et al., 2013; Khater et al., 2020). However, as its input is a fixed density [given by the two user-defined parameters (minimal number of points) per (area defined by the input radius)] it is insensitive to different densities of data points and it is not possible to perform hierarchical clustering (e.g. identifying several dense clusters grouping together to form some larger, higher-order clusters). Data with varying cluster densities can be analysed by OPTICS (Ordering Points to Identify the Clustering Structure) (Ankerst et al., 1999) which returns clusters within their hierarchical structure. Compared to DBSCAN however, OPTICS is computationally demanding, especially for large datasets. These clustering algorithms are implemented in the software suites LAMA and PALMSiever (Pengo et al., 2015; Malkusch and Heilemann, 2016).

Alternatively, clustering can be based on Voronoi polygons (Levet et al., 2015; Voronoi, 1908a, 1908b). Based on the user-defined maximal area of polygons (i.e. dependent on the local density), localizations are assigned to clusters.

### Module 9: Localization Precision and Image Resolution

SMLM imaging is sensitive to experimental conditions, such as the background noise, thermal drift of the sample, labeling strategy (e.g. movement of fluorophore with respect to the target) and imaging procedure (e.g. read-out intensities, camera settings, optics). As a result, the experimental localization precision is normally lower than the theoretically achievable localization precision, which itself scales with the square root of the number of photons emitted by the fluorophore (Mortensen et al., 2010; Turkowyd et al., 2016). Therefore, quantification of the experimental localization precision provides more accurate results, especially concerning the best achievable resolution (i.e. the optimum image resolution is at best twice the localization precision (Nyquist-Shannon sampling theorem) (Nyquist, 1928; Shannon, 1949). Two methods to compute either image resolution or localization precision are described here: Fourier-ring correlation (FRC) (Saxton and Baumeister, 1982; Van Heel et al., 1982; Unser et al., 1987; Banterle et al., 2013; Nieuwenhuizen et al., 2013) and nearest neighbor based analysis (NeNA) (Endesfelder et al., 2014).

### Module 9a: Fourier Ring Correlation (FRC)

**FIGURE 9:**
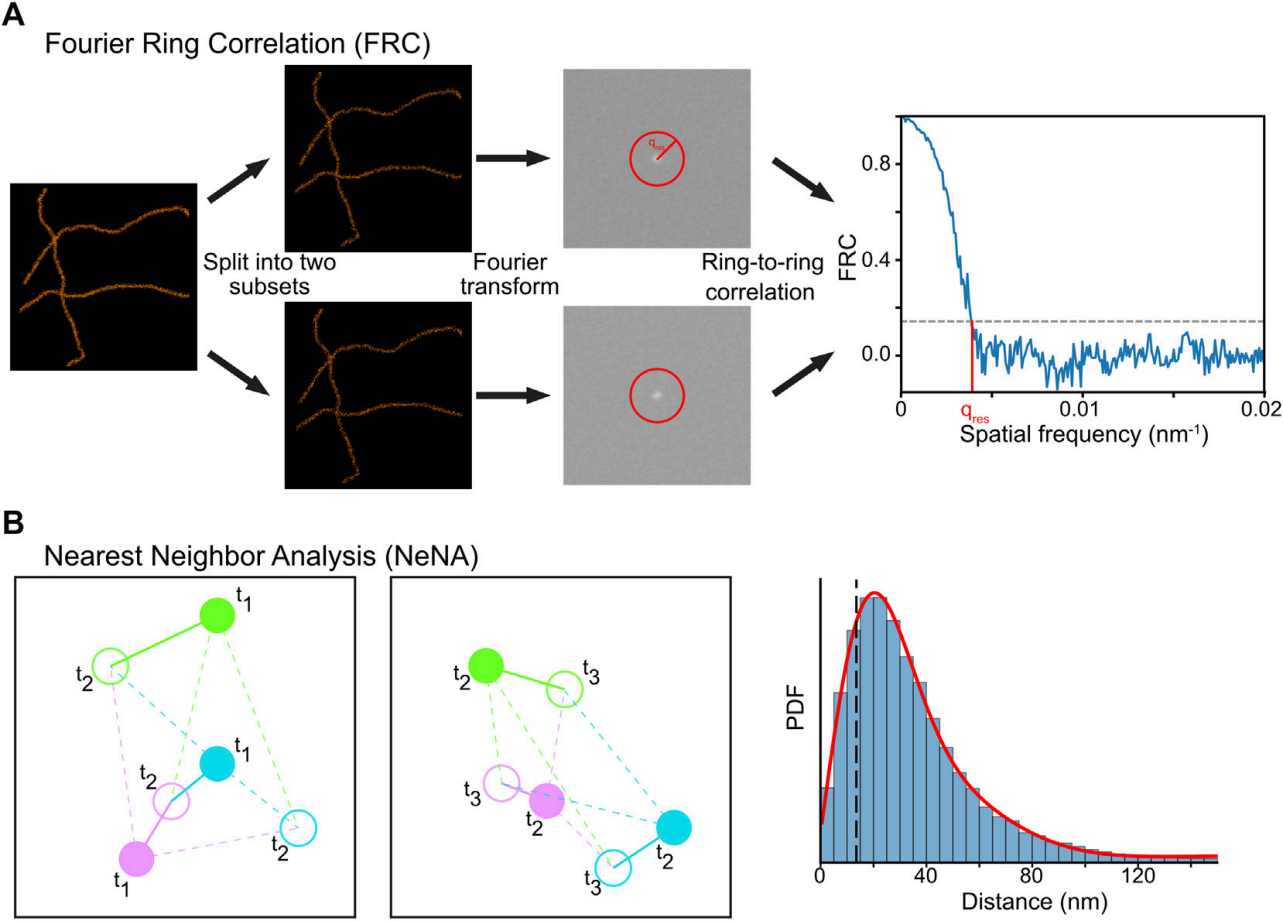
Computational methods to determine resolution in SMLM. **(A)** In FRC, the localization data is split randomly in two subsets, and the correlation of the Fourier transforms of these images at rings with increasing radius is calculated. The resolution of the dataset can then be calculated by determining where the FRC crosses the value of 1/7. **(B)** In NeNA, positions of identical emitters localized in subsequent frames are compared with each other, and the distribution of these distances is fitted with a non-central chi distribution. This fit provides a measure for localization resolution.

FRC is a method used to calculate image resolution by comparing two images taken from the same structure (Saxton and Baumeister, 1982; Van Heel et al., 1982). It can be reasonably applied to SMLM data by splitting the dataset into two halves and assuming that a structure rather than mobile fluorophores are imaged (Banterle et al., 2013; Nieuwenhuizen et al., 2013). The spatial frequency domain spectra of these images are computed via a Fourier transform and are correlated with each other at different distances from the frequency center of the image (Figure 9A). The image resolution is estimated by determining where the FRC value crosses a user-defined value, typically 1/7 ≈ 0.143 (Rosenthal and Henderson, 2003; Banterle et al., 2013; Nieuwenhuizen et al., 2013).

In our module (https://colab.research.google.com/drive/1svyAqyjpdo_hIG8FSCjAmhNznqDq2sFm or https://github.com/Endesfelder-Lab/SMLMComputational, also Supplementary Pseudocode S9), a localization list is randomly split into two arrays, and two images are created (see Module 6). Next, a “distance map” is created with the same size as the two images, which stores the distance to the center of the image. Three required Fourier-transform-based images are then calculated from the two generated images. Finally, the code loops over all distances found in the distance map, and extracts the pixels in the distance map that match this distance. The values in the Fourier-transform-based images belonging to these pixels are then extracted, and the FRC value at this distance is calculated. These distances are plotted in a graph, and the intersection with 1/7 is calculated.

Due to its simplicity, FRC is a widely implemented and used approach (Ovesny et al., 2014; Ries, 2020; Herbert, 2021). However, it can only be used on structural data, as it measures the image similarities via correlation. Also, FRC is sensitive to the non-random division of the data into two bins, e.g. splitting SMLM data into two sub-datasets with only odd or even frames is typically overestimating image resolution as fluorophores commonly fluoresce for more than one consecutive frame. Such effects can be counterbalanced by a correction factor (Nieuwenhuizen et al., 2013). Finally, FRC is affected by the image pixel size used for the visualization and Fourier transform, and additionally requires a structural density that is higher than the localization precision.

### Module 9b: Nearest Neighbor Analysis (NeNA)

The localization precision of a SMLM (sub)dataset can be estimated directly from the localization data using NeNA (Figure 9B) (Endesfelder et al., 2014). As most nearest neighbor events in adjacent frames from non-merged localization data are originating from the same fluorophore which emits photons over several frames, the true distance between these events is zero (assuming a static dataset). To estimate the localization precision, NeNA estimates the apparent jump distances (and thus localization precision) with a non-central chi distribution for two or three dimensions, or a Gaussian for one dimension.

NeNA will only fail if the average lifetime of single fluorophores is (much) less than a single frame, but this is a setting that should be avoided in SMLM experiments to obtain optimal data (Diekmann et al., 2020).

In our module (https://colab.research.google.com/drive/1JbmbEL1XsF6ab4WmL96iLUijN8Tx0LCU or https://github.com/Endesfelder-Lab/SMLMComputational, also Supplementary Pseudocode S9), a two-dimensional localization dataset is loaded, and the distance to the nearest-neighbor in the next frame is calculated (see Module 3). Since the data contains fiducial markers (see Module 4a) and PALM localizations, the localizations are split by their emission intensity. Finally, the jump distance calculated via nearest-neighbor tracking for both PALM- and fiducial marker-localizations are plotted as a histogram, and the distributions are fitted.

## Discussion and Perspective

In the previous modules, we covered the most common computational analysis procedures. However, there are other approaches which can improve the efficiency of the analysis and the quality of results or provide new insights into SMLM data, but are not shown here, either due to their highly specific implementation, niche usage, or computational complexity.

A possibility for any SMLM data analysis is to confine the analysis to user-defined ROIs, i.e. only performing analysis in specific regions, or separating analysis based on these regions (e.g. per cell). This ROI selection can be performed on a variety of measures, but we will exemplify ROI selection via single-cell data analysis, where an outline of the cell is used to separate data analysis. This naturally requires (brightfield or phase-contrast) image data of the cells in addition to SMLM data. Additionally, the cells need to be segmented, either manually or *via* algorithms incorporated in e.g. MorphoLibJ, SplineDist, or Oufti (Legland et al., 2016; Paintdakhi et al., 2016; Berg et al., 2019; Mandal and Uhlmann, 2021). Recently, machine-learning approaches have been created to perform cell segmentation (Ronneberger et al., 2015; Berg et al., 2019; Falk et al., 2019). Machine-learning approaches can be especially powerful, considering it often provides good segmentation performance, and its fast computation could allow for real-time (on-line) segmentation. This opens up avenues for e.g. capturing only subsets of the FoV where cells are present, reducing storage size and downstream computational efforts.

If a structure of interest needs to be resolved with a resolution higher than normally achievable in SMLM, particle averaging is an interesting avenue. In particle averaging, the same structure (e.g. nuclear pore complexes Thevathasan et al., 2019) are visualised many times throughout the FoV. Then, their data is combined, traditionally by mapping the repeated structure to a template structure. Mapping onto a template nevertheless is biased towards the template (e.g. rare but biologically important deviations from the consensus structure will be not detected), is sensitive to insufficient labeling, and requires image generation rather than using the localization data directly (Henderson, 2013). Recently, new particle averaging approaches, namely “all-to-all” registrations and comparing relative localization distances to a model description, have arisen that circumvent these downsides (Curd et al., 2021; Heydarian et al., 2018; Heydarian et al., 2021).

An improvement that concerns all computational analysis procedures is to apply these in real-time; i.e. during the SMLM acquisition rather than during post-processing. However, rapid computations and feedback for online microscope control are non-trivial to achieve. Nonetheless, an increasing number of tools approaches real-time SMLM data analysis and online microscopic feedback (Henriques et al., 2010; Kechkar et al., 2013; Holden et al., 2014; Štefko et al., 2018; Li et al., 2019). These advancements can eventually pave the way for intelligent and fully autonomous live-cell, single-molecule microscopy.

## Data Availability

All data required to run the modules can be obtained from https://github.com/Endesfelder-Lab/SMLMComputational. The data underlying the provided data are available upon reasonable request to the corresponding author.
