# Supplementary material for: Raw Data to Results: A Hands-On Introduction and Overview of Computational Analysis for Single-Molecule Localization Microscopy
Source: Front Bioinform. 2022 Feb 1;1:817254. doi: 10.3389/fbinf.2021.817254 (PMC9580916; doi:10.3389/fbinf.2021.817254)
Supplement: Supplementary file 1 [file DataSheet1.pdf]

Supplementary information

Belonging to:

Raw data to results: a hands-on introduction and overview of computational analysis for single-molecule localization microscopy

Koen J.A. Martens[1,2], Bartosz Turkowyd[1,2,3], Ulrike Endesfelder[1,2,3]

[1]Department of Physics, Carnegie Mellon University, Pittsburgh, Pennsylvania, US
[2]Institute for Microbiology and Biotechnology, Rheinische-Friedrich-Wilhelms-Universität Bonn, Bonn, Germany
[3]Department of Systems and Synthetic Microbiology, Max Planck Institute for Terrestrial Microbiology and LOEWE Center for Synthetic Microbiology (SYNMIKRO), Marburg, Germany

*corresponding author, email: endesfelder@uni-bonn.de

Table of content:

Supplementary Pseudocode 1 – 9

Supplementary Table 1

Supplementary Pseudocode 1
Module 1: Temporal median filter

```
READ microscopy movie
FOR every frame in the movie
      FOR every pixel in the frame
            OBTAIN data range over which the median should be calculated
            CALCULATE the median value of the data range
            SUBTRACT the median value from current pixel/frame in the movie
            and store the value in a new matrix
      ENDFOR
ENDFOR
```

Supplementary Pseudocode 2
Module 2: Localization

```
READ temporally-filtered-movie and raw microscopy movie
FOR every frame in the movie
      CALCULATE difference-of-Gaussian frame to enhance features
      OBTAIN local maxima that have an intensity higher than a set value based on temporally-filtered-movie
      FOR every local maxima found in this frame
            OBTAIN local region-of-interest around this local maximum in raw microscopy movie
            CALL localization_ROI with local region-of-interest RETURNING coordinates
            CALCULATE true position of emitter: add the localization coordinates to the position of the local maximum corner
      ENDFOR
ENDFOR
```

Functions:
Option 1: localization with phasor: also see doi.org/10.1063/1.5005899

```
localization_ROI with local region-of-interest:
      READ local region-of-interest
      CALCULATE 2-dimensional Fourier transform (FT) of local region-of-interest
      EXTRACT first harmonic in x- and y-direction
      CALCULATE position of maximum of first harmonic frequency in x-direction by determining the angle of the harmonic frequency value in a phasor plot
      CALCULATE position of maximum of first harmonic frequency in y-direction by determining the angle of the harmonic frequency value in a phasor plot
      CALL photometry_intensity_calculation with local region-of-interest RETURNING intensity
      RETURN coordinates and intensity

photometry_intensity_calculation with local region-of-interest:
      READ local region-of-interest
      FOR every pixel in the local region-of-interest
            CALCULATE distance from pixel to center of region-of-interest
            IF distance is lower than radius of the region-of-interest
                  ADD pixel to SignalMap
            ELSEIF distance is higher than radius of the region-of-interest
                  ADD pixel to BackgroundMap
            ENDIF
      ENDFOR
      CALCULATE 56th-percentile of intensity of all pixels in BackgroundMap (also see doi.org/10.1016/j.bpj.2016.07.047)
      SUBTRACT the obtained background intensity for every pixel in the SignalMap
      CALCULATE sum of intensity of all pixels in the SignalMap
      RETURN intensity
```

Option 2: localization with 2D-Gaussian fitting (not in module)

```
localization_ROI with local region-of-interest:
      READ local region-of-interest
      SET initial fit parameters
      WHILE fitting is ongoing
            CALCULATE goodness-of-fit between local region-of-interest and 2D Gaussian distribution with current parameters
```

```
    IF fit is good enough
        RETURN fit parameters
    ELSEIF fit is not good enough
        COMPUTE new fit parameters
    ENDIF
ENDIF
```

Supplementary Pseudocode 3
Module 3: Localization merging

```
READ localization data
FOR each localization
      CALCULATE the nearest neighbor in the next 2 frames
      IF the distance to the nearest neighbor is lower than a predefined
      value
            IF this localization is already part of a trajectory
                  SET the trajectory-id of the nearest neighbour to the
                  trajectory-id of the localization
            ELSE (if this localization is not already part of a trajectory)
                  SET the trajectory-id of this localization and the
                  nearest neighbour to the value of tracking-id
                  INCREASE the value of tracking-id by 1
            ENDIF
      ENDIF
ENDFOR
FOR each trajectory
      CALCULATE the mean position weighted by intensity, and sum of
      intensity
      ADD linked molecule to new merged localization list
ENDFOR
FOR each localization that is not part of a trajectory
      ADD the localization to the merged localization list
ENDFOR
```

Supplementary Pseudocode 4
Module 4: Drift correction

Module 4a: Fiducial marker drift correction

```
READ localization data
INITIALISE tracking-id to 1
FOR each localization
      CALCULATE the nearest neighbor in the next frame
      IF the distance to the nearest neighbor is lower than a predefined
      value
            IF this localization is already part of a trajectory
                  SET the trajectory-id of the nearest neighbour to the
                  trajectory-id of the localization
            ELSE (if this localization is not already part of a trajectory)
                  SET the trajectory-id of this localization and the
                  nearest neighbour to the value of tracking-id
                  INCREASE the value of tracking-id by 1
            ENDIF
      ENDIF
ENDFOR
FOR each trajectory
      IF this trajectory has a localization on every frame of the movie
            SET this trajectory as a valid fiducial marker trajectory
      ENDIF
ENDFOR
COMPUTE the average movement of all fiducial marker trajectories on every
frame
FOR each frame
      CALCULATE the average drift with respect to the first frame
ENDFOR
FOR each localization
      SUBTRACT the average drift
ENDFOR
```

Module 4b: Cross-correlation drift correction

```
READ localization data
COMPUTE two datasets that divide localization data correctly
CALCULATE two-dimensional histograms from localization data based on
(Module 6)
FOR each histogram
      CALCULATE the cross-correlation between this histogram and the first
histogram
      CALCULATE the position of maximum intensity of the cross-correlation
      CALCULATE the shift of the maximum position from this histogram to
the first histogram
ENDFOR
CALCULATE the interpolation between the found shifts to encompass all
frames
FOR each frame
      SUBTRACT the drift found on this frame from all localizations on this
frame
ENDFOR
```

Supplementary Pseudocode 5
Module 5: Chromatic aberration correction

```
READ pair-wise datapoints in green and red channel
COMPUTE 2-dimensional affine transformation based on the pair-wise
datapoints
COMPUTE corrected red-channel image based on affine transformation
COMPUTE corrected red-channel localization list based on affine
transformation
```

Supplementary Pseudocode 6
Pseudo-code belonging to Module 6: Image generation

Option 1: Image generation by binning the data and convolution

```
READ localization data
SET pixel size
SET convolution kernel
COMPUTE the two-dimensional histogram from localization data
COMPUTE the convolution of the two-dimensional histogram and convolution
kernel
```

Option 2: Image generation by linear interpolation

```
READ localization data
SET pixel size
FOR each localization
      CALCULATE the corresponding pixel bin
      CALCULATE the distance to the bin center
      CALCULATE the intensity interpolation to neighboring pixels
      COMPUTE the increased intensity to all bins
ENDFOR
```

Option 3: Image generation by Gaussian rendering

```
READ localization data
SET pixel size
FOR each localization
      COMPUTE region of interest (ROI) around emitter center sized 3x
      Gaussian standard deviation
      FOR every pixel in the ROI
            CALCULATE the value of the Gaussian distribution (centered at
            the localization position) in this pixel
            COMPUTE the increased intensity to this pixel
      ENDFOR
ENDFOR
```

Supplementary Pseudocode 7
Module 7: Single particle tracking

```
READ localization data
INITIALISE tracking-id to 1
FOR each localization
      CALCULATE the nearest neighbor in the next frame or the next 2 frames
      IF the distance to the nearest neighbor is lower than a predefined
value
            IF this localization is already part of a trajectory
                  SET the trajectory-id of the nearest neighbour to the
                  trajectory-id of the localization
            ELSE (if this localization is not already part of a trajectory)
                  SET the trajectory-id of this localization and the
                  nearest neighbour to the value of tracking-id
                  INCREASE the value of tracking-id by 1
            ENDIF
      ENDIF
ENDFOR
FOR each trajectory
      FOR each localization in the trajectory
            COMPUTE all jump distances
      ENDFOR
      COMPUTE the mean jump distance of this trajectory
ENDFOR
```

Single particle tracking quantification: Jump Distance (JD) histogram

```
COMPUTE a histogram of all jump distance values
COMPUTE the fit of this histogram with one or multiple Rayleigh
distributions
```

Single particle tracking quantification: Mean Jump Distance (MJD) histogram

```
COMPUTE a histogram of all mean jump distance values (possibly weighted by
trajectory length)
COMPUTE the fit of this histogram with one or multiple Gaussian
distributions
```

Supplementary Pseudocode 8
Module 8: Localization clustering (DBSCAN)

```
READ localization data
FOR each localization
      COMPUTE the nearest neighbor in all frames, store the distance and
      the nearest neighbor index
      IF the localization has at least a predetermined minimum number of
neighbours
            SET core-status to TRUE
            SET cluster-status to TRUE
      ENDIF
ENDFOR
FOR each localization
      IF the localization is not core
            IF the localization has at least 1 core neighbour
                  SET cluster-status to TRUE
            ENDIF
      ENDIF
ENDFOR
INITIALISE label-id to 1
FOR each localization
      IF the localization is core
            IF the localization does not have an assigned label
                  SET the localization label to the label-id
                  CALL recursion loop
            ENDIF
      ENDIF
      INCREASE label-id by 1
ENDFOR

RECURSION LOOP:
IF the localization is cluster
      IF the localization does not have an assigned label
            SET the localization label to the label-id
            IF the localization is core
                  FOR all neighbours of this localization under a
      predetermined distance
                        CALL recursion loop
                  ENDFOR
            ENDIF
      ENDIF
ENDIF
```

Supplementary Pseudocode 9
Module 9: resolution/accuracy determination

Module 9a: Fourier Ring Correlation (FRC)

```
READ drift-corrected localization data
FOR every localization
      DETERMINE to which of two data arrays the localization will be
randomly added
END
COMPUTE two images from the two localization data arrays (Module 6)
COMPUTE the normalization of both images (total sum of each image should be
1)
COMPUTE Fourier Transform (FT) of both images
COMPUTE f1f2*: FT of image 1 multiplied by the conjugate of FT of image 2
COMPUTE f1^2: squared FT of image 1
COMPUTE f2^2: squared FT of image 2
INITIALISE a map of the same size as the images
CALCULATE the integer (rounded) distance of every pixel in the map to the
center of the map
FOR every distance found in the distance map
      COMPUTE the pixels that correspond to the ring of this distance
      COMPUTE the sum of the values in f1f2* in this pixelated ring and
store in an array
      COMPUTE the sum of the values in f1^2 in this pixelated ring and
store in an array
      COMPUTE the sum of the values in f2^2 in this pixelated ring and
store in an array
      COMPUTE the FRC value at this distance: divide the value of (the sum
      of the values in this ring of) f1f2* by sqrt(f1^2*f2^2)
ENDFOR
COMPUTE the resolution by finding where the FRC values cross the line 1/7
```

Module 9b: NeNA resolution determination

```
READ drift-corrected localization data
FOR all localizations
      CALCULATE the nearest neighbor in the next frame
      IF the distance to the nearest neighbor is lower than a predefined
value
            ADD the distance to an ongoing list of distances
      ENDIF
ENDFOR
COMPUTE a histogram of all values in the list of distances
CALCULATE the best fit of the data with the NeNA formula (noncentral Chi
distribution)
```

Supplementary Table 1

Overview of software or plug-ins that contain (methods similar to) the modules in this manuscript.

**Software/plug-in**	Module 1: Temporal median image filtering	Module 2: Localization	Module 3: Localization merging	Module 4a: Fiducial marker drift correction	Module 4b: Cross-correlation drift correction	Module 5: Chromatic aberration correction	Module 6: Image generation	Module 7: Single particle tracking	Module 8: Localization clustering	Module 9a: Fourier-ring correlation structural resolution	Module 9b: NeNA localization precision	Reference(s)
**Our manuscript**	x	x	x	x	x	x	x	x	x	x	x	[This manuscript]
**ZOLA-3D**		x	x		x		x					(Aristov et al., 2018)
**Icy**		x						x	x			(de Chaumont et al., 2012)
**QuickPALM**		x					x					(Henriques et al., 2010)
**GDSC SMLM**		x	x	x	x		x	x	x	x		(Herbert, 2021)
**SMALL-LABS**	x	x					x	x				(Isaacoff et al., 2019; Martens et al., 2021)
**FTM2**	x											(Jabermoradi et al., 2021)
**SR-Tesseler**									x			(Levet et al., 2015)
**LAMA**						x			x		x	(Malkusch and Heilemann, 2016)
**DEEPSTORM-3D**		x										(Nehme et al., 2020)
**ThunderSTORM**		x	x	x	x		x					(Ovesny et al., 2014; Martens et al., 2018)
**Radial Symmetry**		x										(Parthasarathy, 2012)
**PALMsiever**				x	x		x		x			(Pengo et al., 2015)
**SMAP**		x			x	x	x			x		(Ries, 2020)
**Picasso**		x		x	x	x	x		x			(Schnitzbauer et al., 2017)
**DECODE**		x					x					(Speiser et al., 2021)
**Trackmate**		x						x				(Tinevez et al., 2017)
**RapidSTORM**		x	x	x			x					(Wolter et al., 2012)
